# ILF2: a multifaceted regulator in malignant tumors and its prospects as a biomarker and therapeutic target

**DOI:** 10.3389/fonc.2024.1513979

**Published:** 2024-12-13

**Authors:** Tonglin Sun, Xi Li, Yi Zhang, Bingwen Zou, Yan Zhang

**Affiliations:** ^1^ Department of Medical Oncology, Cancer Center, West China Hospital, Sichuan University, Chengdu, Sichuan, China; ^2^ Lung Cancer Center/Lung Cancer Institute, West China Hospital, Sichuan University, Chengdu, Sichuan, China; ^3^ General Practice Ward/International Medical Center Ward, General Practice Medical Center, West China Hospital, Sichuan University, Chengdu, Sichuan, China; ^4^ Department of Thoracic Surgery and Institute of Thoracic Oncology, West China Hospital of Sichuan University, Chengdu, Sichuan, China

**Keywords:** ILF2, NF45, cancer, prognosis, biomarkers, nuclear protein

## Abstract

Interleukin enhancer binding factor 2 (ILF2), formerly called nuclear factor 45 (NF45), is widely expressed in normal human tissues. ILF2 often binds to interleukin enhancer binding factor 3 (ILF3) and regulates gene expression in several ways, participating in multiple DNA and RNA metabolism pathways. Recent studies have shown that ILF2 expression is significantly upregulated in esophageal cancer, lung cancer, gastric cancer, and other malignant tumors, which can promote tumor development and tumor cell proliferation, affect the cell cycle, and induce epithelial-mesenchymal transition. ILF2 expression is closely related to tumor cell migration and invasion, neo-angiogenesis, and patient prognosis. ILF2 is expected to become a biomarker for the early diagnosis of patients with tumors and assessing their prognosis. This article reviews the role of ILF2 in malignant tumors and its related mechanisms.

## 1 Introduction

**Figure 1:**
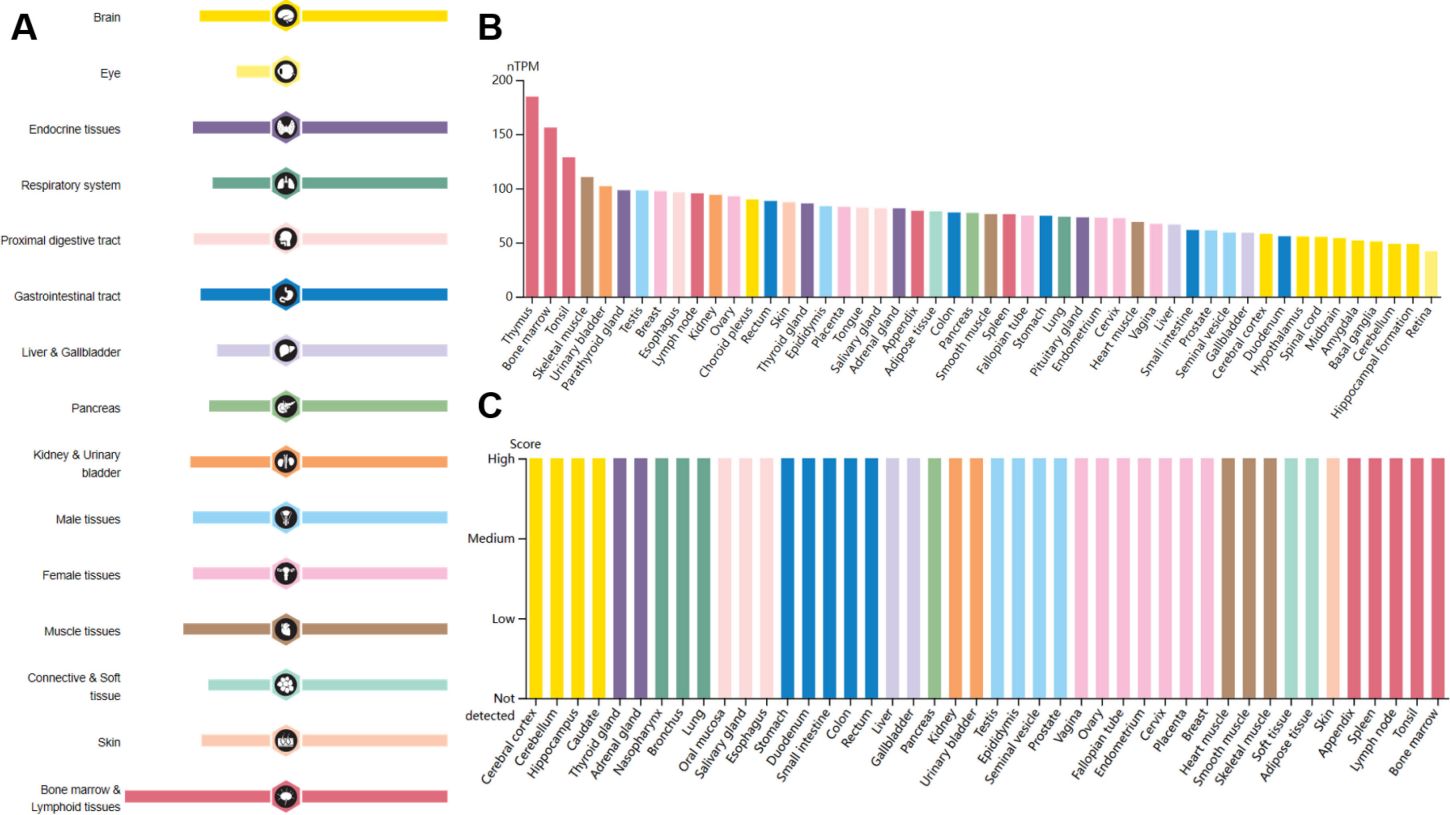
RNA and protein expression profile of ILF2 in human organs and tissues. Data from the Human Protein Atlas. **(A)** The summary of ILF2 mRNA and protein expression in human organs and tissues; **(B)** ILF2 mRNA expression summary in different human organs and tissues based on consensus dataset; **(C)** ILF2 protein expression summary in different human organs and tissues.

Interleukin enhancer-binding factor 2 (ILF2/NF45) is a ubiquitously expressed nuclear protein that plays critical roles in various cellular processes. It is particularly abundant in the testis, brain, and kidney tissues (**Figure 1**) (1). The *ILF2* gene is located on human chromosome 1q21.3 and was initially identified as a subunit of the nuclear factor of activated T cells (NFAT), a key transcription factor complex essential for the expression of interleukin 2 (*IL2*) in T cells (2, 3). ILF2 often forms a heterodimeric complex with interleukin enhancer-binding factor 3 (ILF3/NF90/NF110), which together regulate gene expression at multiple levels (4–7). ILF2 and ILF3 are multifunctional proteins involved in various aspects of DNA and RNA metabolism, including DNA binding, transcriptional regulation, DNA damage response, RNA transcription and translation, mRNA splicing, and microRNA (miRNA) biogenesis (4–7). Through these activities, ILF2 profoundly impacts cellular functions such as proliferation, differentiation, and stress response. Moreover, ILF2 has been implicated in immune responses, inflammation, viral replication, and autoimmune diseases, highlighting its significance in maintaining cellular homeostasis and responding to external stimuli (8–10).

Recent studies have brought attention to the role of ILF2 in cancer biology. Accumulating evidence indicates that *ILF2* expression is significantly upregulated in various malignant tumors, including cervical (11), breast (9), pancreatic (12), lung (13), liver (14), and esophageal (15) cancers. This overexpression suggests that *ILF2* may function as an oncogene, promoting tumor growth, survival, and metastasis. For example, ILF2 has been shown to enhance tumor cell proliferation by regulating the cell cycle and inhibiting apoptosis (16). It also facilitates tumor progression by promoting cell migration, invasion, and angiogenesis (17).

Malignant tumors continue to pose a significant global health challenge, with high morbidity and mortality rates despite advances in traditional treatments such as surgery, radiotherapy, and chemotherapy. These conventional therapies often have limited efficacy and are associated with considerable side effects, emphasizing the need for novel therapeutic strategies. A deeper understanding of the molecular mechanisms underlying tumor development and progression is crucial for identifying new therapeutic targets and improving patient outcomes. In this context, ILF2 has emerged as a potential key player in tumor biology due to its multifaceted roles in gene regulation and cellular function. However, the precise mechanisms by which ILF2 contributes to tumorigenesis and cancer progression remain incompletely understood. While some studies have begun to elucidate the pathways involving ILF2, such as its interaction with ILF3 and regulation of miRNA processing (18), there remains a lack of comprehensive insight into its functions across different tumor types.

Given the critical role of ILF2 in various cellular processes and its upregulation in multiple cancers, a thorough review of its biological functions, expression patterns, and regulatory mechanisms in malignant tumors is warranted. Therefore, this review aims to synthesize current research findings to highlight the significance of ILF2 in tumorigenesis. By examining the existing literature, we seek to understand how ILF2 contributes to cancer development and explore its potential as a prognostic marker and therapeutic target. We hope this examination will provide new directions and ideas for cancer research and therapy, ultimately contributing to improved treatment strategies and patient survival.

## 2 Structure and functions of ILF2

ILF2 contains a domain associated with zinc fingers (DZF), which is also found in ILF3, spermatid perinuclear RNA-binding protein (STRBP/SPNR), and zinc finger RNA-binding protein (ZFR). The DZF domain is the only folded region in ILF2 and is flanked by N- and C-terminal domains rich in arginine/glycine and glutamate sequences, respectively. It resembles the zinc finger structure of template-free nucleotidyltransferase RNA-modifying enzymes, promoting protein dimerization and RNA binding. ILF2 can form complexes with other DZF-containing proteins involved in RNA metabolism (19).

As an RNA-binding protein, ILF2 participates in mitosis and DNA damage repair, promotes embryonic stem cell proliferation and differentiation, enhances mRNA stability, and negatively regulates miRNA production (20, 21). It interacts with other RNA-binding proteins such as Y-box binding protein 1 (YBX1/YB1), adenosine deaminase RNA specific (ADAR/ADAR1), and heterogeneous nuclear ribonucleoproteins to regulate pre-mRNA stability and alternative splicing, influencing DNA damage response and genome stability (22). Overexpression of *ILF2* due to 1q21 amplification in multiple myeloma correlates with disease progression and resistance to DNA-damaging agents (23, 24). The ILF2/ILF3 complex enhances mRNA stability and upregulates mRNA expression during mitosis (25). Knockdown studies suggest this complex may compete with staufen double-stranded RNA binding proteins 1 (STAU1) and 2 (STAU2) in regulating mRNAs during mitosis, affecting the cell cycle. Overexpression of *ILF2* and *ILF3* can inhibit the conversion of primary miRNAs into precursor miRNAs, decreasing mature miRNA levels (18). In addition, ILF2 plays a crucial role in regulating IL2, a 15.5 kDa glycosylated globular protein important for T-cell growth (26). Along with ILF3, X-ray repair cross-complementing 5 (XRCC5/Ku80), and X-ray repair cross-complementing 6 (XRCC6/Ku70), ILF2 is involved in *IL2* chromatin remodeling and gene expression in activated T cells (27). ILF2/ILF3 heterodimers bind specifically to the distal antigen-recognition response element (ARRE) site in the *IL2* enhancer (2). Overexpression of *ILF2* increases IL2 activity and ARRE/NF-AT luciferase activity, suggesting it induces *IL2* expression through the ARRE/NF-AT promoter sequence (1). ILF2 and ILF3 also co-regulate *IL2* mRNA transport and stability (28).

## 3 Role of ILF2 in malignant tumors

**Figure 2:**
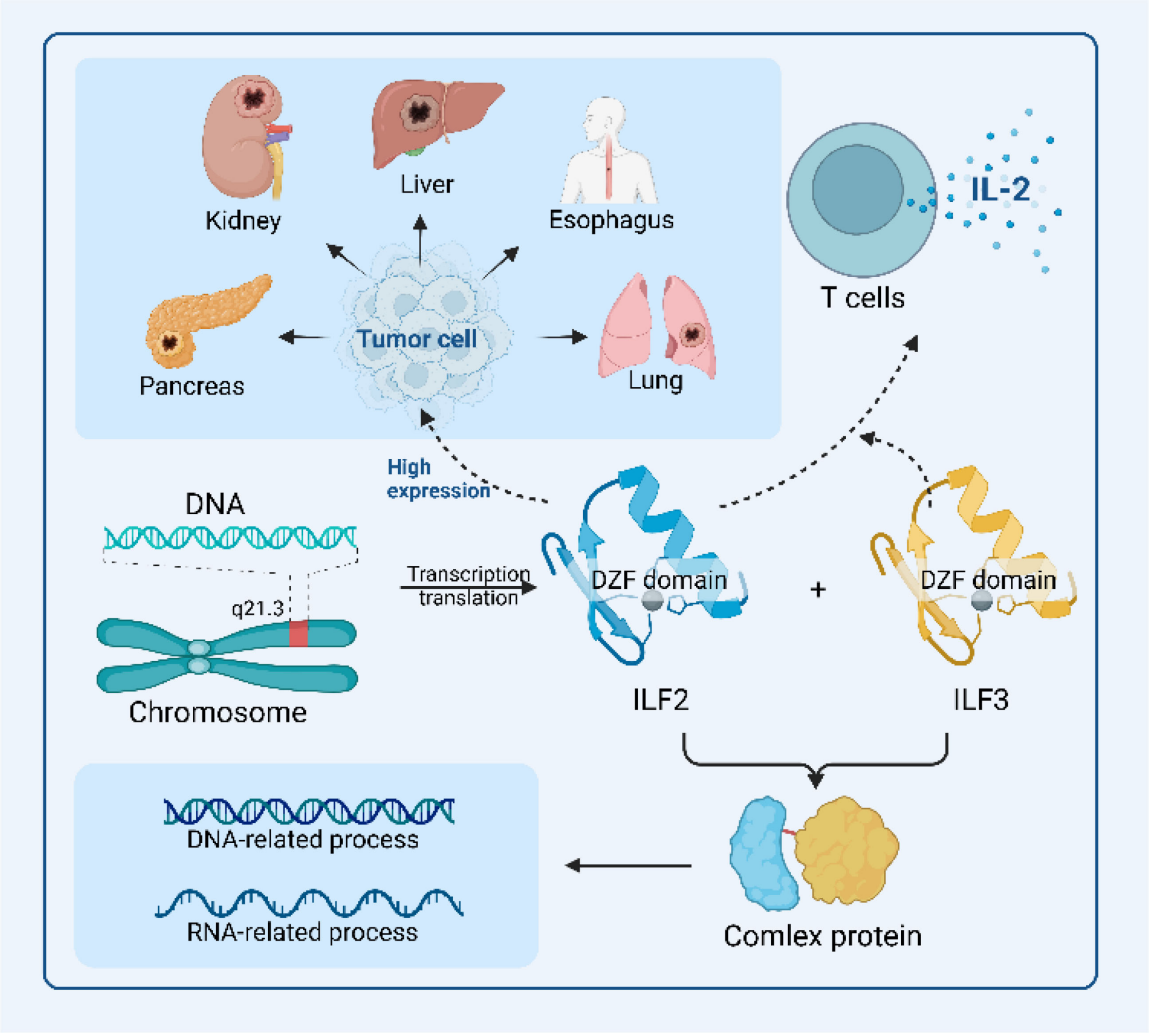
Overview of ILF2’s functional roles in malignant tumor environments. The schematic diagram highlights the structural features of ILF2, particularly its DZF domain, which facilitates its binding to ILF3 to form a functional protein complex, which is integral to DNA-related and RNA-related processes, including transcription and translation. In combination with ILF3, ILF2 also regulates *IL2* expression, thereby promoting T cell growth and proliferation. The elevated expression of *ILF2* across various biological and pathological contexts underscores its mechanistic roles and therapeutic potential in malignant tumorigenic environments.

This section explores the multifaceted role of ILF2 in malignant tumors, focusing on its expression patterns, oncogenic activities, and its influence on cell proliferation, survival, cell cycle progression, migration, invasion, and angiogenesis. Furthermore, we discuss the potential of ILF2 as a prognostic biomarker, diagnostic marker, and therapeutic target in cancer treatment. The overview of ILF2's role is shown in **Figure 2**.

### 3.1 *ILF2* expression in malignant tumors

*ILF2* expression is significantly upregulated in various malignant tumors compared to normal tissues. Studies have shown high *ILF2* expression in esophageal squamous cell carcinoma (ESCC) (16), hepatocellular carcinoma (HCC) (14), non-small-cell lung cancer (NSCLC) (29), pancreatic ductal adenocarcinoma (PDAC) (12), gastric cancer (30), breast cancer (9), and gliomas (31). The overexpression of *ILF2* is often associated with advanced tumor stages, poor differentiation, and increased tumor aggressiveness.

### 3.2 ILF2 as an oncogene in tumor development

ILF2 plays an oncogenic role in the development of malignant tumors by promoting cell proliferation, inhibiting apoptosis, and contributing to tumorigenesis. In gliomas, *ILF2* expression correlates with higher World Health Organization grades, suggesting a role in glioma progression (31). In HCC, *ILF2* overexpression promotes cell proliferation by stabilizing cAMP-response element-binding proteins (CREB) and activating the epidermal growth factor receptor (EGFR)/protein kinase B (AKT) signaling pathway (32). In small-cell lung cancer, ILF2 promotes cell proliferation and maintains mitochondrial homeostasis (29).

### 3.3 ILF2 promotes tumor cell proliferation and survival

ILF2 enhances tumor cell proliferation and survival through various mechanisms. *ILF2* knockdown inhibits the expression of cell cycle regulators such as cyclin E1 (*CCNE1*/*CCNE*) and proliferating cell nuclear antigen (*PCNA*) in PDAC, thereby reducing cell proliferation (12). In HCC, *ILF2* overexpression upregulates anti-apoptotic proteins B-cell leukemia/lymphoma 2 (BCL2) and baculoviral IAP repeat containing 2 (BIRC1) while downregulating pro-apoptotic proteins BCL2-associated X apoptosis regulator (BAX) and BCL2 family apoptosis regulator BOK (BOK), inhibiting apoptosis (14).

### 3.4 ILF2 influences cell cycle progression

ILF2 affects cell cycle progression by regulating the expression of cell cycle-related proteins. In ESCC, ILF2 influences the G_0_/G_1_ to S phase transition, implying a role in cell cycle regulation (16). In NSCLC, *ILF2* knockdown leads to cell cycle arrest, inhibiting cell proliferation (13). In PDAC, *ILF2* overexpression contributes to the G1-S phase transition, promoting cell cycle progression (12).

### 3.5 ILF2 promotes tumor cell migration and invasion

ILF2 enhances tumor cell migration and invasion, contributing to tumor metastasis. In ESCC, ILF2 activates the TIAM Rac1-associated GEF 1 (TIAM1)/Rac family small GTPase 1 (RAC1) signaling pathway, promoting cell growth and invasion (33). In pancreatic cancer cells, *ILF2* overexpression increases migration and invasion by regulating genes involved in epithelial-mesenchymal transition, such as upregulating vimentin (*VIM*) and fibronectin 1 (*FN1*) while downregulating cadherin 1 (*CDH1*/E-cadherin) (17). Ex vivo experiments in nasopharyngeal carcinoma showed that the ILF2/ILF3 complex interacts with differentiation antagonizing non-protein coding RNA (DANCR) to stabilize the hypoxia-inducible factor 1 subunit alpha (*HIF1A*) mRNA, enhancing cell migration and invasion (34).

### 3.6 ILF2 and tumor angiogenesis

ILF2 indirectly promotes tumor angiogenesis by upregulating pro-angiogenic factors. Guarnerio et al. (35) demonstrated that circular RNAs can interact with nuclear RNA-binding proteins ILF2 and ILF3 to activate the ILF2/ILF3 complex, increasing the expression of pro-angiogenic and growth factors, thereby promoting neovascularization in mesenchymal tumors.

### 3.7 ILF2 as a prognostic biomarker in malignant tumors

High *ILF2* expression is associated with poor prognosis in patients with malignant tumors. In ESCC, high *ILF2* expression correlates with lower disease-free survival (DFS) and overall survival (OS) (33). In gastric cancer, high *ILF2* expression is associated with deeper invasion, lymph node metastasis, advanced TNM stage, and poor differentiation, affecting both DFS and OS (30). In breast cancer, high *ILF2* expression is associated with an increased risk of recurrence, distant metastasis, and death (9). Therefore, ILF2 may serve as a prognostic biomarker in various cancers.

### 3.8 ILF2 and HPV *E6* gene expression

ILF2 promotes the expression of the human papillomavirus (HPV) *E6* gene, which is crucial in cervical carcinogenesis (11). The ILF2/ILF3 complex positively regulates HPV *E6* expression. Thus, knocking down ILF2/ILF3 leads to increased tumor protein p53 (TP53) levels and induces apoptosis in cervical cancer cells (36). Therefore, ILF2 may be a potential therapeutic target in HPV-related cancers.

## 4 ILF2 as a diagnostic marker and therapeutic target

The overexpression of *ILF2* in various malignant tumors highlights its potential as a biomarker for early diagnosis and as a target for therapeutic intervention. The significant difference in *ILF2* expression between tumor tissues and adjacent normal tissues suggests that measuring ILF2 levels could aid in the detection and prognosis of cancers.

Chung et al. (37) analyzed gene expression profiles in colorectal cancer and identified *ILF2* among 13 genes that could serve as early diagnostic markers. By comparing the gene expression in adenomas and colorectal cancer tissues, they found that *ILF2* gene expression was more closely associated with the progression from adenoma to carcinoma than with inflammatory bowel disease. This indicates that ILF2 could be instrumental in detecting colorectal cancer at an early, more treatable stage. Ni et al. (13) found that *ILF2* expression in NSCLC was markedly upregulated in tumor tissues compared to adjacent non-tumorous tissues. The increased ILF2 levels were closely associated with the degree of tumor differentiation, clinical stage, and the proliferation marker MKI67. Kaplan-Meier survival analysis revealed that patients with high *ILF2* expression had shorter OS. Furthermore, multivariate Cox regression analysis identified *ILF2* expression as an independent prognostic factor for NSCLC. These findings suggest that ILF2 could serve as a diagnostic marker and a prognostic indicator, helping clinicians assess disease progression and patient outcomes. Yin et al. (30) observed that high *ILF2* expression in gastric cancer was correlated with deeper tumor invasion, lymph node metastasis, advanced TNM stage, and poor differentiation. Patients with elevated ILF2 levels had shorter DFS and OS. The study concluded that *ILF2* expression could be used to predict recurrence risk and overall prognosis in gastric cancer patients. Beyond serving as a biomarker, ILF2’s role in tumor progression makes it a promising therapeutic target. Knocking down *ILF2* expression in various cancer cell lines has demonstrated inhibitory effects on tumor growth and metastasis. For example, *ILF2* knockdown in HCC led to reduced cell proliferation and increased apoptosis by regulating the balance of pro-apoptotic and anti-apoptotic proteins (14). Silencing *ILF2* expression in PDAC inhibited cell cycle progression and decreased the expression of cell cycle regulators like cyclin E1 (CCNE) and proliferating cell nuclear antigen (PCNA), hindering tumor cell proliferation (12). ILF2 also plays a role in key signaling pathways crucial for tumor survival and metastasis. In ESCC, ILF2 activated the TIAM1/RAC1 signaling pathway, enhancing cell growth and invasion (33). Thus, targeting ILF2 could disrupt these pathways, providing a novel approach to inhibit tumor progression. Moreover, ILF2 may influence the effectiveness of existing treatments. Jin et al. (9) found that although high *ILF2* expression was associated with poorer prognosis in breast cancer, patients with elevated ILF2 levels responded better to anthracycline/paclitaxel neoadjuvant chemotherapy. This suggests that *ILF2* expression could potentially guide treatment decisions, helping personalize therapy based on a patient’s likelihood of responding to specific chemotherapeutic agents. Furthermore, ILF2 is implicated in the regulation of HPV gene expression in cervical cancer, particularly the *E6* and *E7* oncogenes, which are critical in cervical carcinogenesis (36). Thus, knocking down *ILF2* expression resulted in decreased E6 and increased TP53 levels, leading to apoptosis of cervical cancer cells. This indicates that *ILF2* could be targeted to disrupt HPV oncogene expression, offering a therapeutic strategy against HPV-related cancers.

Given these multifaceted roles, ILF2 is a valuable target for therapeutic intervention. Developing drugs or genetic therapies that inhibit *ILF2* expression or function could suppress tumor growth, induce apoptosis, and reduce metastasis. Additionally, monitoring ILF2 levels could enhance the early detection of cancers, allowing for timely and effective treatment.

## 5 Discussion

ILF2 plays an oncogenic role in malignant tumors, and its expression is significantly upregulated in tumor tissues and closely related to patients’ histological grading, TNM stage, MKI67 level, and prognosis, suggesting that ILF2 plays an important role in tumor cell development, migration, and invasion. Therefore, detecting ILF2 expression has potential clinical applications, such as predicting tumorigenesis, early diagnosis, targeted therapy, and assessing prognosis. However, few studies have examined the molecular mechanisms and signaling pathways of ILF2 in related tumors, and little is known clinically about the mechanism of ILF2 in malignant tumors. Alternatively, upstream and downstream genes can be screened through gene libraries, especially key genes significantly related to ILF2, to explore the roles of these key genes and ILF2 in tumorigenesis and development to assess whether they can become proto-oncogenes, biomarkers, or anti-tumor therapeutic targets. However, further studies are still needed to confirm this.

## 6 Conclusion

High *ILF2* expression correlates with poor prognosis, advanced tumor stage, and lower OS in patients, suggesting its potential as a prognostic biomarker. Moreover, ILF2’s role in regulating the stability of mRNAs and miRNAs positions it as a crucial factor in gene expression regulation within tumor cells. These findings have opened new avenues for considering ILF2 as a potential therapeutic target in cancer treatment. Despite these advances, several research gaps remain. The precise molecular mechanisms and signaling pathways through which ILF2 contributes to tumorigenesis are not fully elucidated. The interactions between ILF2 and other oncogenic factors and its role in specific signaling cascades require further investigation. Additionally, most studies have been preclinical, highlighting the need for clinical trials to validate ILF2 as a reliable biomarker and therapeutic target.
